# Croatian Version of the Short Assessment of Health Literacy for Spanish Adults (SAHLSA-50): Cross-Cultural Adaptation and Psychometric Evaluation

**DOI:** 10.3390/healthcare10010111

**Published:** 2022-01-06

**Authors:** Harolt Placento, Božica Lovrić, Zvjezdana Gvozdanović, Nikolina Farčić, Tihomir Jovanović, Jelena Tomac Jovanović, Lada Zibar, Nada Prlić, Štefica Mikšić, Nina Brkić Jovanović, Robert Lovrić

**Affiliations:** 1 General Hospital Našice, 31500 Našice, Croatia; harolt.placento@obnasice.hr (H.P.); zvjezdana.gvozdanovic@obnasice.hr (Z.G.); 2 Faculty of Medicine, Josip Juraj Strossmayer University of Osijek, 31000 Osijek, Croatia; bozicalovric@gmail.com (B.L.); nfarcic@mefos.hr (N.F.); ladazibar@gmail.com (L.Z.); nadaprlic26@gmail.com (N.P.); 3 Nursing Institute “Professor Radivoje Radić”, Faculty of Dental Medicine and Health Osijek, Josip Juraj Strossmayer University of Osijek, 31000 Osijek, Croatia; ihi.pakrac@gmail.com (T.J.); aanelejj@gmail.com (J.T.J.); smiksic@fdmz.hr (Š.M.); 4 General County Hospital Požega, 34000 Požega, Croatia; 5 Department of Surgery, University Hospital Centre Osijek, 31000 Osijek, Croatia; 6 General Hospital Pakrac and Hospital of Croatian Veterans, 34550 Pakrac, Croatia; 7 High School Pakrac, Matije Gupca 59, 34550 Pakrac, Croatia; 8 Department for Nephrology, Clinical Hospital Merkur, 10000 Zagreb, Croatia; 9 Faculty of Medicine, University in Novi Sad, 21000 Novi Sad, Serbia; nina.brkic-jovanovic@mf.uns.ac.rs

**Keywords:** health literacy, test instrument, short assessment of health literacy for Spanish adults (SAHLSA-50), community health

## Abstract

(1) Background: Short Assessment of Health Literacy for Spanish Adults (SAHLSA-50) was originally designed for Spanish-speaking regions, and translations validated for several languages. The aim of the study was to adapt and verify the psychometric characteristics of SAHLSA-50 in the Croatian context; (2) Methods: The cross-sectional study included 590 respondents from the general population older than 18 years of age. Health literacy was measured by two scales: SAHLCA-50 and the Croatian version of the Newest Vital Sign screening test (NVS-HR), which was used as a measure of concurrent validity. Subjective Health Complaints (SHC) and Satisfaction with Life Scale (SWLS) questionnaires were also used to assess convergent validity; (3) Results: Internal consistency reliability of SAHLCA-50 was high and corresponds to the findings of the authors of the original research. The Cronbach alpha coefficient for SAHLCA-50 version was 0.91. The correlation of SAHLCA-50 with the NVS-HR test speaks in favor of concurrent validity. Correlation between health literacy and SHC speaks for convergent validity, just as was expected, while correlation with life satisfaction was not observed; (4) Conclusions: The SAHLCA-50 test can be a good and quick tool to assess health literacy of the adult population in the Croatian language. HL can affect the health and quality of life of the individual and the wider community.

## 1. Introduction

The concept of health literacy (HL), which appeared for the first time in the mid-1970s [1], is considered to be an important behavioral determinant of health, involving many components [2]. There are numerous definitions of HL. We can define it as “personal, cognitive, and social skills that determine an individual’s ability to access, understand, and use information to improve and maintain health” [3]. HL is most often defined through three levels: functional, interactive, and critical. At the functional level, a patient is expected to be able to understand and follow simple health instructions and steps. At the interactive level, a patient should have developed communication and social skills to control his/her health in collaboration with professionals. The third level is critical, and the patient can critically analyze health information as well as actively participate in the treatment and the resolution of health problems [4]. Thus, we may consider a person to be health-literate when they apply health concepts and information in some new situations and participate in dialogues related to health, medicine, scientific knowledge, and cultural beliefs [5]. Research of the term HL has shown that low HL levels were associated with poorer treatment outcomes, higher costs, and dissatisfaction of healthcare providers as well as the users themselves. On the contrary, a highly health-literate person takes responsibility for their own health, health of the family, and ultimately their community [5,6,7]. As indicated by research conducted in the United States of America, it often happened that the level of HL in patients was overestimated [8]. HL is globally considered as one of the fundamental elements of improving and promoting health needed for successful sustainable development by 2030 [9]. The concept of health literacy is multidimensional. The literature review revealed seven distinct dimensions of HL: functional literacy, factual and procedural knowledge, awareness, a critical dimension, an affective dimension, and attitudes [10]. Functional literacy is the best-established dimension of health literacy. To date, ‘gold standard’ measures of health literacy concentrate on this dimension, measuring word recognition, comprehension, and numeracy skills in the medical setting [10].

There are more than 200 instruments used as a measure of HL in the world [11,12,13,14,15,16], e.g.,: Test of Functional Health Literacy in Adults (TOFHLA) [17], Short Assessment of Health Literacy for Spanish Adults (SAHLSA-50) [18], Rapid Estimate of Adult Literacy in Medicine (REALM) [19], Health Literacy Questionnaire (HLQ) [20], All Aspects of Health Literacy Scale (AAHLS) [21], Demographic Assessment of Health Literacy (DAHL) [22], and others. These instruments differ in terms of their content, method of application, the time required for completion, analysis, and processing of results, predicted sample size, and level of reliability and validity [23]. The most commonly used assessment instruments for HL are Test of Functional Health Literacy in Adults (TOFHLA) and Rapid Estimate of Adult Literacy in Medicine (REALM). However, the TOFHLA and REALM instruments have shown some implementation difficulties (e.g., limited applicability in the lower-education populations, long lead times, different translation difficulties, etc.) [24]. Furthermore, one of the most commonly used tests is the Short Assessment of Health Literacy for Spanish Adults (SAHLSA-50). SAHLSA-50, by authors Lee et al. in its original form, was designed and written in Spanish [18] and thus far has been translated and validated in several languages [11,24,25,26], and serves to examine functional health literacy, which is the primary focus of this study. Furthermore, the motives and reasons for choosing the instrument (SAHLSA-50) for this study are primarily determined by the research question and purpose of the study and a number of other elements (instrument availability, theoretical and practical basis of instrument content, good metric characteristics and reliability, population, manner and level of complexity of instrument application, material resources, available time, etc.) [27]. Therefore, SAHLSA-50 is considered as an instrument suitable for procedures of translation and backtranslation; it estimates objective functional health literacy; it is easily used and interesting, with good metric characteristics, suitable for different groups of respondents (healthy and sick population, mentally ill, people with lower level of education and lower literacy, vulnerable and minority groups, the elderly, etc.) [18,24].

SAHLSA-50 requires respondents to read aloud a list of medical terms and associate each term with another word of similar meaning in order to demonstrate their ability to read and understand [18]. Literature available to us indicates that a complete psychometric analysis of the popular SAHLSA-50 test does not exist in the Croatian context, except for the language validation conducted by Berlančić et. al, 2020 [28]. For the performance of relevant intercultural comparative studies in the field of health literacy, instruments should be universally applicable in their existing form [27,29]. This area of health literacy is still not sufficiently explored in this part of Europe [30]. Given that many studies suggest that health literacy can affect the health and quality of life of the individual and the wider community [31,32,33,34,35,36], it is important to ensure timely, systematic, and continuous assessment of HL levels in healthy and sick populations [18]. In Croatia, there is currently a need for a reliable and valid instrument that assesses HL levels and can be applied universally. The only way to achieve global synergy in comparing scientific and professional data on HL is a sufficient number of studies based on validated instruments. This psychometric analysis of SAHLSA-50 seeks to contribute to and assist in theoretical explanation, scientific interpretation and a generally better understanding of the functional HL construct. Furthermore, psychometric confirmation of the validity of this instrument has a practical contribution because it provides a reliable instrument for future scientific and professional studies in Croatia and intercultural comparisons of results. Furthermore, comparative studies can significantly advance the study of the HL phenomenon in different cultural contexts and different strata of society.

Finally, to obtain an answer to the research question of whether SAHLSA-50 is a suitable and reliable instrument for measuring HL in the Croatian context, the main research objective of this study was to validate the SAHLSA-50 test and to verify its metric characteristics.

## 2. Materials and Methods

### 2.1. Study Design and Sampling

A cross-sectional study was conducted from April to December 2018. A total of 590 respondents (42.4% male; median age 52, from 18 to 91 years of age) participated in the study (Table 1). Criteria for inclusion of the subjects were modified according to the recommendations of the authors of the original version of the SAHLSA-50 test [18]. The study included subjects older than 18 years of age, who spoke Croatian, and who were able to participate in the study due to their health condition, without symptoms and signs of cognitive disorders, or drug or alcohol intoxication [18].

The process of sample recruitment was based on: (a) satisfactory diversity criteria concerning the general characteristics of the respondents (age, gender, education and profession, place of residence, habitation of urban or rural areas, marital status, social status, religion, etc.) and (b) voluntary participation of respondents in studies. Seven researchers, the authors of this article (employed in four different health facilities) invited potential respondents to participate in the study during working hours in their health facilities using appropriate situations, times, and places (clinics, offices/administrations, patient waiting rooms, and other formal and informal spaces within their health facilities). Thus, the study participants were outpatients and their accompaniment from different areas, their visitors to hospital patients (family, relatives, friends), non-medical staff (outsourcing), technical staff and insurance, carriers, delivery people, etc. The study did not include health professionals in these four institutions. Determination of sample size was conducted regarding the literature agenda where it is considered to have a minimum of 10 participants per scale item, and thus the sample of 590 ensures an adequate sample size [37]. There are certain risks of bias, as is well known, which are sometimes almost impossible to avoid in the sampling process especially in the social sciences and humanities [38]. In this study, such biases were convenient sampling and the inability to know the characteristics of respondents who refused to participate.

### 2.2. Study Tools

The Croatian version of SAHLSA-50 [28] was used in the study to examine its psychometric values. According to the recommendations of the authors of the original version of the SAHLSA-50 test, the Croatian version of the instrument is called the Short Assessment of Health Literacy for Croatian Adults (henceforth SAHLCA-50). Furthermore, for the purpose of additional verification/confirmation of the concurrent and convergent validity of the SAHLCA-50 instrument, three additional instruments were used: Newest Vital Sign (NVS) [39], Subjective Health Complaints (SHC) [40], and Instrument Satisfaction with Life Scale (SWLS) [41]. The introductory part of the instrument included questions for collecting demographic and other general data (e.g., gender, age, education, place of residence, and marital status).

#### 2.2.1. Instrument Short Assessment of Health Literacy for Croatian Adults (SAHLCA-50)

We used the SAHLCA-50 test [28], a previously translated and linguistically adapted Croatian version of the original SAHLSA-50 in Spanish [18]. Based on recommendations given by Francisco et al. [42] and Wild et al. [43], the questionnaire was adapted to meet cross-cultural equivalence. In the first step, the procedure of translating from Spanish into Croatian was carried out by three independent translators with the help of a Spanish-language teacher (native Croatian speaker). These translators had never encountered the instrument used in this research before. The second step was a back-translation into Spanish conducted by one independent translator (fluent bilingual native speaker of Spanish living in Croatia). Subsequently, the authors and translators analyzed the translations and developed a pre-final version based on all the translations and back-translations performed as described above. Finally, several randomly selected volunteers examined the instrument, checking each item for precision and clarity of the question asked [28]. Volunteers were persons selected by the same method as the respondents and their socio-demographic characteristics were completely consistent with the respondents [27]. After voluntarily checking the clarity and comprehensibility of the written instructions and the content of the instrument, they gave feedback to the researchers, but did not participate in the study as respondents. The test consists of 50 medical terms, accompanied by one related and one unrelated term serving as a distractor, and the “I do not know” option. The purpose of the “I do not know” answer was to reduce the random guessing of the answers. Lee et al. [18] deliberately chose easier terms as answers because their goal was to enable respondents with lower levels of education also to understand the questions. The task of the respondent was to select an answer that accurately described the medical term. All correct answers were scored with 1, and incorrect answers or the answer “I do not know” were scored with 0, so the possible range of results ranged from 0 to 50.

#### 2.2.2. Instrument Newest Vital Sign (NVS)

As the second HL test, and for the purpose of verifying the concurrent validity of the SAHLCA-50 instrument, we used the Croatian version of the Newest Vital Sign (NVS-HR) [44], which represents a reliable Croatian version of the original NVS-UK instrument [2] and is used to measure HL as the same construct measured by SAHLCA-50. The implementation procedure consisted of showing the nutritional declaration to the examinees during the examination. After the respondent had read it, the examiner asked seven questions of an objective type that the respondent should have answered without using the aids to calculate the answer. Answers were scored as correct or incorrect, and the possible range of scores ranged from 0 to 6, whereby a higher score indicated greater HL. The seventh question was actually a semi-question, and was asked in the case that the answer to the sixth question was too general, but still somewhat correct. If the respondent answered the seventh question correctly, one point was added to the sixth question. In case the respondent did not answer the seventh question correctly, the sixth question was scored zero points. The reliability of the type of internal consistency in this study was 0.77, which was similar to the value obtained in the original paper [39]. A factor analysis was also performed using the principal components procedure and two significant factors were obtained. Since all particles had saturations greater than 0.61 on the first factor, the one-dimensional structure was retained, and the resulting factor explained 46.96% of the variance of the variables.

#### 2.2.3. Instrument Subjective Health Complaints (SHC)

The Croatian-validated version of SHC [45] was used in this study to further examine the convergent validity of the SAHLCA-50 instrument, as relevant studies suggest an association between HL and somatic and psychological complaints [46]. It consisted of 29 items, and the task of the respondent was to estimate the frequency of occurrence of various symptoms on a scale from 1 (never) to 5 (always) in the last year. Eriksen et al. found five factors in their work, which, however, could be grouped into one general health factor. The overall result was a simple linear combination of responses to all claims, and a higher score on this questionnaire indicated more frequent onset of symptoms [40]. The reliability of the internal consistency type in the original study was 0.82, while in this study the measured value was 0.9.

#### 2.2.4. Instrument Satisfaction with Life Scale (SWLS)

Since research indicates that HL variables are significant predictors of individual components of quality of life [36], in order to further confirm the convergent validity of the SAHLCA-50 instrument, the Croatian-validated version of the Satisfaction With Life Scale (SWLS) was used in this study [47]. The SWLS [41] is intended to measure the general cognitive assessment of life satisfaction. It consisted of five particles, and the task of the examinee was to assess the degree of agreement with statements on a Likert scale from 1 (I strongly disagree) to 7 (I strongly agree). The overall result was a simple linear combination of answers to all statements, whereby a higher score indicated greater life satisfaction. The reliability of the internal consistency type in the original study was 0.87, while in this study the measured value was 0.84.

### 2.3. Data Collection Procedures

The survey was conducted from April to December 2018 by the authors of the article. Data were collected from May to October 2018, through questionnaires distributed among the general population. Respondents were informed about the purpose of the research both in written form and verbally. Respondents confirmed their consent to participate in the survey by signing an informed consent form. Participation in the study was offered to 600 respondents, of whom 10 refused to participate, making a final sample of 590 respondents and an extremely unexpectedly high and relevant response rate of 98.3% [48].

Researchers collected data by conducting testing with each participant individually “face-to-face”, while ensuring the privacy and anonymity of respondents. According to the authors’ recommendations for the use of the original instrument SAHLSA-50 [18], the examiner used fifty plasticized cards with the subjects. Each numbered card contained a written key medical term (so-called ‘main word’) and additionally written/offered three terms/explanations (one correct answer, one incorrect answer, and one “I do not know” answer). The respondents read and verbalized the main word from the card, analyzed the offered answers, and, after thinking, verbalized one answer that they considered correct, i.e., complementary to the parent word, or gave the answer “I do not know”. Respondents’ response time was not limited. The same answering method applied to all 50 cards. The researcher recorded the verbal answers of the respondents on the prepared questionnaire [18].

After that, according to the recommendations of the authors of the NVS-HR questionnaire, the respondents read the terms from the plasticized nutritional declaration of the food item (ice cream), after which they calculated the nutritional values and answered the examiner’s questions. The use of aids to calculate the answers (calculator, pen, and paper) was not allowed. The researcher recorded the (in)accuracy of the answers on the questionnaire. There was no time limit for responding, and the average response time was 20 min. After the HL tests with the pen-and-paper method, the respondents filled out a questionnaire on sociodemographic data (gender, age, education, place of residence, and marital status) (Table 1) and SHC and SWLS questionnaires, which lasted on average 10 min.

### 2.4. Data Analysis

The analysis of the collected data was performed using the statistical package IBM SPSS 23. In order to examine the factor structure of SAHLCA-50 on the Croatian sample, we performed Confirmatory Factor Analysis (CFA) and the maximum likelihood method to verify the fit of the one-factor model described in the previous studies [49]. The following indices were used: the penalizing function (χ^2^/degrees of freedom [df]), with values lower than 3 indicating good fit; the comparative fit index (CFI), which ranges from 0 to 1 and with a minimum good fit value of 0.90; and the standardized root mean square residual (SRMR) index, with values lower than 0.08 indicating a good fit [50].

The receiver operating characteristic (ROC) curve was used to determine the optimal cut-off value of SAHLCA-50 for the categories health-literate/health-illiterate. The area under the curve (AUC), and 95% confidential interval, sensitivity, and specificity were reported for the complete sample. The ROC curves were interpreted as the probability that the estimated interval values can adequately discriminate health-literate from health-illiterate respondents (0.5 is chance discrimination, 1.0 is perfect discrimination). To determine the optimal cut-off value, we used the point on the ROC curve closest to (0,1) and the Youden J statistics [51]. The Youden index (J) was calculated as [sensitivity + specificity/1], and the point with shortest distance value from the point (0,1) was calculated as [(1 × sensitivity)/2 + (1 × specificity)/2]. The significance value was set at *p* < 0.05.

The descriptive data used were mean, standard deviation, range, asymmetry, flatness, and median and interquartile range. Reliability of the internal consistency type using the Cronbach’s alpha coefficient was calculated. Pearson’s correlation coefficient was used to examine the correlation of the obtained results, in order to check the convergent and divergent validity, and due to deviation from the normal distribution, bootstrapping was used on 2000 samples, which was also used in the remaining analyses. The t-test for independent samples and one-way analysis of variance (ANOVA) were used to examine differences between groups. A Tukey post-hoc test was used to examine differences between individual groups after ANOVA.

### 2.5. Ethical Considerations

The conduct of the research was previously approved by the ethics committees of all healthcare institutions in which the research was conducted (approval numbers: R1-4979-4/2018; 01-168/3-2018; 01-679/5-2018; 02-7/53-1/2-8-2018). This study was conducted according to the Helsinki Declaration of Human rights, participation was voluntary, and the participants were informed of the right to withdraw from the study without any consequences. Informed consent was attached to each questionnaire, as well as consent for voluntary participation in the research.

## 3. Results

The median age of the respondents was 52 years, and the interquartile range (IQR) was from 37 to 63, with a minimum of 18 to a maximum of 91 years of age. Basic demographic data are shown in Table 1.

With the purpose of verifying the one-factor structure of the questionnaire, a CFA and the maximum likelihood method were conducted. The Bartlett sphericity test and the Kaiser–Meyer–Olkin Test (KMO) were used as standard measures to evaluate sampling adequacy and factor data validity. The Bartlett’s test of sphericity indicated that the results are not a random correlation (*p* < 0.00). A KMO test of 0.82 as a measure of sample adequacy showed that sampling was adequate for the variables in the model as well as for the model as a whole, thus meeting the conditions for conducting factor analysis of the questionnaire.

The CFA computed with the obtained results showed that all of the scale’s items can be grouped into a single dimension and according to a common factor associated with all the items. The KMO measure of sampling adequacy was 0.823. The chi-square test, used as a significance test of the minimized discrepancy function during model fitting, was statistically significant (*p* < 0.001), CFI = 0.67, CMIN/df = 2.93, and the RMSEA was 0.057. These results show that the unidimensional model has a good fit for the observed data. SRMR total was 0.05, which is also indicative of a good fit.

Table 2 shows the percentage of correct and incorrect answers for each item as well as the loading factor of each item. The items that were responsible for the higher number of incorrect answers (more than 50%) were item 24 “Jaundice” (72%), item 25 “Pap smear” (61.9%), item 13 “Constipation” (59.8%), item 37 “Herpes” (55.7%), and item 45 “Colitis” (54.7%) (Table 2). The range of correlations of the responses for individual statements with the total score was from 0.25 to 0.57, which was satisfactory.

Table 3 shows the descriptive data with the reliability of the internal consistency type for SAHLCA-50, NVS-HR, SHC, and SWLS tests.

In order to verify the concurrent and convergent validity of SAHLCA-50, correlation of the respective test with other measures was calculated, the results of which are shown in Table 4.

The results of the respondents at SAHLCA-50 with regard to sociodemographic characteristics are presented in the next section (Table 5).

Women were more health-literate than men, and respondents living in the city were more health-literate than respondents living in the countryside. When examining differences between education levels, post-hoc analysis using the Tukey test found that there were mutual differences between all groups of respondents (*p* = 0.01, resp.), except between the group with Bachelor and the group with Master’s degrees. Respondents with a higher level of education were more health-literate. When it comes to marital status, post-hoc analysis found that only widows were less health-literate than every other category of the respondents (*p* = 0.01, resp.). Correlation between HL and age showed that older respondents achieved lower results (r = −0.33; *p* < 0.01).

Receiver operating characteristic (ROC) curve analysis was used in order to determine the cut-off points on the SAHLCA-50, which is good for distinguishing adequate from inadequate health literacy. The entry literacy categories were entered through the NVS-HR questionnaire, whereby the respondents with a score from 0 to 3 in this questionnaire were classified into the group of those who do not have adequate health literacy, while those who had a score from 4 to 6 in the NVS- HR questionnaire were classified into the category of health-literate.

**Figure 1:**
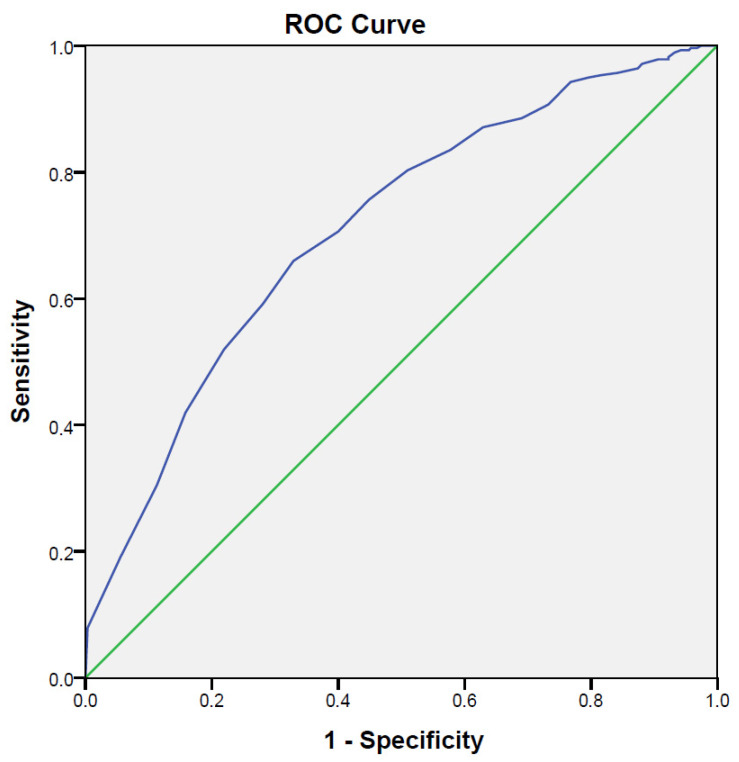
Receiver operating characteristic (ROC) curves of the SAHLCA-50.

ROC curves showed that an SAHLCA-50 value of 42 was the determinant for reasonable expectation of the existence of adequate HL, with sensitivity of 75%, specificity of 70%, AUC of 0.71 (95%CI, 0.76–0.83, *p* < 0.001), and with highest Youden index of 0.59 (Figure 1).

## 4. Discussion

Nowadays, much health-related information is available. It is thus important to have basic medical knowledge in order to better understand the instructions of health professionals, and consequently follow them [52]. Low HL levels represent a significant public health challenge throughout Europe, where one in every three to almost one in every two Europeans may not be able to understand essential health-related material [53]. The research presented in this paper was conducted with the aim of validating HL test SAHLCA-50 and checking its metric characteristics because HL is also an important factor in understanding health-related information [53].

The discussion of the results presented below is presented in a structured way through the analysis of the basic parameters of assessing the usability of the instrument: (a) the structure of the construct and the respondents’ answers, (b) the reliability of the instrument, and (c) competitive and convergent validity in the Croatian sample.

When we considered the structure of the construct being estimated by the instrument, the results of the conducted CFA indicate the existence of a one-factor model that showed good fit for the observed data through linear modeling, which is in line with the results of another study [49]. The grouping of questions around one factor clearly indicates that HL is a one-dimensional construct that does not have subscales, which is confirmed by the literature descriptions of the theoretical basis of HL [18]. Such results are important for the theoretical and scientific definition of the health-literacy construct and indicate the relevance of the SAHLCA-50 instrument for testing the HL construct.

In the next step, the structure of the obtained factor was analyzed in terms of the achievement of the examinees and the factor loadings of each individual item. With an insight into the structure of factors, this analysis verified the contribution of the item and the possible need to shorten the scale, as was the case in other studies [26,54,55]. Thus, the researchers in the mentioned research marked and concluded that certain questions can be excluded in future research, because such questions do not have sufficient variability and do not ensure adequate discrimination/screening of respondents from the aspect of health-literate or -illiterate. Given the percentages of correct and incorrect responses of respondents in this study, and in the context of the psychometric properties of SAHLCA-50, there was no need to remove questions from the questionnaire as was the case in other studies [26,54,55]. The process of reaching this conclusion consisted of semantic review of the items in the test and analysis of factor loadings of each item. Using semantic review of the items in the test, we concluded that respondents scored worse when it came to terms that are more similar to common professional medical terms (e.g., jaundice) compared to everyday terms (e.g., flu).

In this study, there were only six questions to which more than 96% of respondents gave correct answers: Pill 96.3%, Medication 96.8%, Flu 96.9%, Eye 96.9%, Bowel 96.9%, and Diabetes with a maximum of 98.3% correct answers. The fact that these six terms had the highest percentage of correct answers can be explained by the fact that they refer to the main and known organs/parts of the human body, to forms of drugs used by a large number of people, and to common and modern diseases. Furthermore, these terms are very often used in everyday medical and non-medical communication, including the public media. Additionally, some original Spanish terms had to be changed to make the content of the questionnaire and the use of terms in SAHLCA-50 more appropriate to the Croatian linguistic context [28]. This can be noted as a weakness of this study, but at the same time a recommendation for future similar comparative studies. Furthermore, only one question was answered incorrectly by more than 60% of the respondents (Pap smear 69.1%), indicating that all 50 questions in SAHLCA-50 were discriminatory enough and there was no need to remove any of the questions. All factor loadings were statistically significant and the questions that had the highest item loading on the HL factor and contributed the most to the HL assessment were questions number 10 Caffeine (0.57), and 15 Abnormal (0.51), while question number 4 Flu (0.21) had the lowest item loading.

The reliability of the type of internal consistency of the questionnaire was also confirmed and corresponded to the findings of the author of the original paper [18]. The value of the Cronbach alpha coefficient (α = 0.91) indicates an extremely high level of reliability of SAHLCA-50 for testing the HL construct, with also high values of the Cronbach alpha coefficient for the other three instruments used in parallel (NVS-HR = 0.78, SHC = 0.90, SWLS = 0.84). The high level of reliability of SAHLSA-50 is also shown by other studies [18,49,56], which indicates the intercultural applicability of this instrument.

As the next step, to examine the concurrent validity of SAHLCA-50, the Pearson correlation coefficient with the NVS-HR scale (as confirmed) was calculated and it was found that those two measures were interrelated, indicating that they measured a similar construct. The convergence of SAHLCA-50 was supported by an association with the NVS-HR test, which also measured HL. Thus, the results suggest that these two instruments are extremely similar in terms of the construct of HL which they measure, which further justifies the use of NVS-HR in the ROC curve as a measure of binary input to determine which of the subjects is health-literate/illiterate. Such a high level of association between these two constructs further suggests the validity of SAHLCA-50 for HL measurement. This result was in a way expected, as SAHLSA-50 and NVS significantly correlated with the TOFHLA-S questionnaire [57]. It should be emphasized again that NVS-HR was a more complex and demanding test of HL for both respondents and researchers themselves, since it required logical–mathematical analysis in solving tasks.

To measure the convergent validity, two comparative instruments were applied: a measure of Subjective Health Complaints (SHC) and a measure of life satisfaction (SWLS). SAHLCA-50 correlated with SHC questionnaire significantly negatively, thus confirming the convergent validity of the scale. On the other side, it was found that there was no significant association with HL measured by SAHLCA-50 and a measure of life satisfaction, while other researchers found lower correlation [58]. In contrast, a significant correlation was found between the NVS-HR and the SWLS instrument for measuring life satisfaction. This confirms that, despite the fact that NVS-HR and SAHLCA-50 measures are very similarly defined constructs of HL, there are still differences between the two instruments. The SAHLCA-50 probably measures a part of the HL construct that is not saturated with the concept of life satisfaction. Furthermore, as previously mentioned, NVS-HR required certain logical and mathematical processes from the test subjects, while SAHLCA-50 was a simpler instrument to apply while focusing on informing/familiarizing the subjects with aspects of human health.

In our research, women were more health-literate than men, while some researchers did not find these differences [56,59,60], and respondents living in the city were more health-literate than respondents living in the countryside. When examining differences between education levels, there were differences between all groups of respondents, except between those with Bachelor’s and Master’s degrees, and respondents with a higher level of education were more health-literate. When it comes to marital status, it was found that only widows were less health-literate than other respondents. Research conducted in eight European countries also indicates that a number of demographic characteristics such as age, gender, marital status, emigrant status, and other socioeconomic factors such as personal income affect HL. These studies also indicate variability of HL among the surveyed countries, as well as the connection between HL and health outcomes [30]. The importance of HL in following medical instructions and taking medication were proven by numerous papers [35,61,62].

The results of the ROC curve indicate that the cut-off score for SAHLCA-50 is at 42 determinants with a sensitivity of 75% and a specificity of 70%, suggesting a satisfactory and acceptable binary outcome for the HL construct and instrument functionality in the Croatian context. It can be concluded that the respondents generally achieved good results on this test. Lee et al. [18] determined in their work that a score greater than 37 indicated health-literate respondents, and 24.8% of the respondents in their study scored less than 37. In this study, the result which indicates health illiteracy was less than 42. Such a high cut-off score was deliberately set to raise the sensitivity level of the SAHLCA-50 scale. In this way, an effort was made to ensure the most reliable selection of health-literate and -illiterate respondents and to minimize the risk of creating an “unrealistic image” in terms of false HL-literate respondents. This score provides a reasonable level of possible false-positive or false-negative results with a relatively satisfactory percentage of probability and reliability of detection of health-(ill)literate persons. A review of the literature shows that researchers thus far have not used a cut-off score of 42 and that it was first recorded in this study. These results are not surprising, regarding the existence and impact of different cultural specificities as well as differences in formal and non-formal education and healthcare systems of the general population. Therefore, the results of this first analysis of the psychometric values of SAHLCA-50 in the Croatian context should be understood as a useful initial step for further testing and international comparative analysis of the HL construct.

### Study Limitations

There are several limitations to this study that should be considered. First, the manner and location of data collection and the process of recruiting respondents, which additionally resulted in an extremely high response rate of respondents. Such selection processes that use a convenience sample generate a considerable selection-bias risk and it was very difficult for us to avoid this completely. However, the presence of potential respondents at given locations in relation to the time factor and their sociodemographic characteristics was completely unpredictable, thus minimizing the bias of the research. Second, it should be mentioned that in this research there is an evident lower representation of respondents from more extremely rural areas with a possibly poor flow of health information or people who are significantly distanced from the services of the health system. It is also important to note that the structure of the final sample indicates a lower percentage of respondents with a university degree and respondents who are not married, which should certainly be considered when interpreting and generalizing the results of this study. An important fact that justifies us is the fact that the educational structure of the sample corresponds to the general educational structure in the Republic of Croatia, which is dominated by the population with secondary education [63]. Third, as noted in the paper’s discussion of the limitation regarding specific terms in SAHLCA-50, there are always risks of less adequate translations of terms from the original instrument, although all prescribed translation and back-translation rules have been followed. All of the above weaknesses need to be upgraded to future similar comparative studies.

## 5. Theoretical, Scientific, and Practical Implications

This study contributes to the theoretical and scientific explanation and deeper understanding of the functional HL dimension. The results point to the one-dimensionality of the HL construct in the Croatian context, which has a national, but also a significant global contribution to understanding and upgrading the theoretical framework on which HL is based. Furthermore, this encourages intercultural conceptual questioning of functional HL as the “gold standard” in relation to the other six dimensions of HL [10]. Such theoretical analyses and international comparisons are extremely important in understanding the scientific basis of the HL construct.

From a practical point of view, the Short Assessment of Health Literacy for Croatian Adults (SAHLCA-50) could be a very effective and sustainable instrument that will enable continuous research of the level of functional HL of healthy and sick people in all segments of the health system in the Republic of Croatia. Furthermore, SAHLCA-50 can be used in combination with other research approaches to examine possible correlations between HL levels and various individual and contextual factors (e.g., age, gender, education level, cultural factors, IQ, emotional intelligence, etc.). In the immediate healthcare process, through timely and effective assessment of HL levels using SAHLCA-50, health professionals can obtain useful information on the level of HL of healthcare users and thus adapt methods and strategies of communication, education, and other supportive methods aimed at healthcare users. This can be noted as a weakness of this study but at the same time a recommendation for future similar comparative studies.

Furthermore, this study should be an effective incentive for future scientific studies primarily targeting specific individuals in the population (patients with various acute and chronic diseases, the mentally ill, vulnerable groups in the population, national minorities, etc.). Future analyses should also focus on the formation of predictor models within which it is possible to detect the contribution of HL to quality of life and prevention of previously described problems of people with lower HL levels (e.g., poorer treatment outcome, higher healthcare costs, dissatisfaction of healthcare providers and customer dissatisfaction, etc.).

## 6. Conclusions

After validation and verification of the SAHLCA-50 test, we can conclude that the test showed good metric characteristics and it is easy to apply and interpret. We can use it as a short triage test to examine HL in the general population. Besides the ease of application, SAHLCA-50 primarily focuses on informing/familiarizing the respondents with aspects of human health and does not require additional intellectual, logical–mathematical abilities of the respondents. Performing the test does not require additional training of medical staff, which makes it more accessible in use for various purposes. Some shortcomings were the easy tasks in the test; however, with the determination of a higher cut-off level, an optimal binary outcome was achieved to examine the HL level in the Croatian context. The SAHLCA-50 test for Croatian-speaking respondents can be a good and a fast tool for HL examination of adults in Croatia. With this tool, HL can be examined in a particular community and knowledge of the current situation gained. According to Emee Vida Estacio [64], partnership in health promotion can be significantly improved with the help of vital elements such as shared vision, mutual trust, respect, and openness to sharing and communication. Quality of communication and understanding between the patient and the healthcare staff can be raised. This has the potential to improve the quality of care and reduce healthcare costs of service in a particular community.

## Figures and Tables

**Table 1:** Sociodemographic characteristics of respondents (N = 590).

Variable	Category	N	%
Gender	Male	250	42.4
	Female	340	57.6
Education	No education	33	5.6
	Primary school	110	18.6
	Secondary school	328	55.6
	Bachelor’s degree	61	10.3
	Master’s degree	58	9.8
Residence	Rural	295	50.2
	Urban	293	49.8
Marital status	Unmarried	105	17.8
	Married	378	64.1
	Divorced	36	6.1
	Widow/widower	71	12

**Table 2:** Display of response items and item factor loadings (N = 590).

SAHLSA-50 (Lee SYD, 2006)	SAHLCA-50 (Berlančić, 2020)	% of Correct Answers	% of Incorrect Answers	Item Factor Loading
1. Prostate	1. Prostata	78.1	22.9	0.45
2. Occupation	2. Zaposlenje	89.2	10.8	0.39
3. Menstrual	3. Menstruacijsko	93.7	6.3	0.38
4. Flu	4. Gripa	96.9	3.1	0.25
5. Notify	5. Upozoriti	91.5	8.5	0.36
6. Meals	6. Jelo	93.9	6.1	0.37
7. Alcoholism	7. Alkoholizam	92.2	7.8	0.41
8. Fat	8. Masnoća	95.3	4.7	0.32
9. Asthma	9. Astma	95.1	4.9	0.49
10. Caffeine	10. Kofein	85.6	14.4	0.57
11. Osteoporosis	11. Osteoporoza	83.7	6.3	0.48
12. Depression	12. Depresija	92.9	7.1	0.33
13. Constipation	13. Konstipacija	40.2	59.8	0.41
14. Pregnancy	14. Trudnoća	93.6	6.4	0.48
15. Incest	15. Incest	68.6	31.4	0.46
16. Pill	16. Pilula	96.3	3.7	0.29
17. Testicle	17. Testis	81.2	18.8	0.47
18. Rectal	18. Rektalno	71.4	28.6	0.55
19. Eye	19. Oko	96.9	3.1	0.33
20. Irritation	20. Iritacija	77.1	22.9	0.44
21. Abnormal	21. Abnormalno	83.1	16.9	0.51
22. Stress	22. Stres	95.1	4.9	0.48
23. Miscarriage	23. Spontani pobačaj	92.4	7.6	0.48
24. Jaundice	24. Ikterus	28	72	0.27
25. Pap smear	25. Papanicolau	38.1	61.9	0.33
26. Impetigo	26. Osip	94.2	5.8	0.36
27. Directed	27. Naznaka	86.4	3.6	0.44
28. Attack	28. Napad	93.4	6.4	0.34
29. Menopause	29. Menopauza	91.2	8.8	0.42
30. Appendix	30. Slijepo crijevo	95.3	4.7	0.41
31. Behavior	31. Ponašanje	95.1	4.9	0.33
32. Nutrition	32. Prehrana	86.6	3.4	0.27
33. Diabetes	33. Dijabetes	98.3	1.7	0.34
34. Syphilis	34. Sifilis	69	31	0.50
35. Inflammatory	35. Upala	90.3	9.7	0.35
36. Hemorrhoids	36. Hemoroidi	81.7	18.3	0.44
37. Herpes	37. Herpes	44.7	55.3	0.42
38. Allergic	38. Alergija	82.7	17.3	0.37
39. Kidney	39. Bubreg	95.9	4.1	0.34
40. Calories	40. Kalorije	88.1	11.9	0.43
41. Medication	41. Lijek	96.8	3.2	0.31
42. Anemia	42. Anemija	82	18	0.43
43. Bowel	43. Crijeva	96.9	3.1	0.41
44. Potassium	44. Kalij	75.6	24.4	0.47
45. Colitis	45. Kolitis	45.3	54.7	0.46
46. Obesity	46. Pretilost	93.2	6.8	0.48
47. Hepatitis	47. Hepatitis	78.8	21.2	0.41
48. Gallbladder	48. Žučni mjehur	84.7	15.3	0.39
49. Seizure	49. Epilepsija	94.9	5.1	0.45
50. Arthritis	50. Artritis	66.3	33.7	0.44

rIT—Item Total correlation (r)—correlation of each statement with the total result.

**Table 3:** Descriptive statistics and basic characteristics of the distribution of applied scales.

	Mean	SD	Range	Asymmetry	Flattery	Cronbach α
SAHLCA-50	41.58	7.23	6–50	−1.61	3.44	0.91
NVS-HR	3.29	2.02	0–6	−0.16	−1.16	0.78
SHC	63.8	16.01	29–116	0.48	−0.46	0.90
SWLS	24.12	6.46	5–35	−0.46	−0.06	0.84

**Table 4:** Correlation between applied scales.

	SAHLCA-50	NVS-HR	SHC	SWLS
SAHLCA-50	1	0.46 **	−0.12 **	0.07
NVS-HR		1	−0.17 **	0.13 **
SHC			1	−0.24 **
SWLS				1

** *p* < 0.01.

**Table 5:** Average values on SAHLCA-50 and tests of the difference in relation to sociodemographic characteristics.

**Variable**	**Category**	**Mean**	**SD**	***t***	***p***
Gender	Male	40.24	7.75	−3.9	0.01
	Female	42.56	6.67		
Residence	Rural	40.25	7.35	−4.53	0.01
	Urban	42.92	6.89		
**Variable**	**Category**	**Mean**	**SD**	***F* (*df*)**	***p***
Education	No education	30.85	10.15	52.33 (4.589)	0.01
	Primary school	37.56	7.93		
	Secondary school	42.41	6.09		
	Bachelor’s degree	45.39	4.4		
	Master’s degree	46.56	3.62		
Marital status	Unmarried	42.22	6.41	15.24 (3.589)	0.01
	Married	42.29	6.41		
	Divorced	42.58	7.53		
	Widow/widower	36.32	9.9		

## Data Availability

All data generated analyzed during the current study are available from the corresponding author on reasonable request.
